# Examining intervention mechanisms of action using mediation analysis within a randomised trial of a whole-school health intervention

**DOI:** 10.1136/jech-2018-211443

**Published:** 2019-02-05

**Authors:** Chris Bonell, Elizabeth Allen, Charles Opondo, Emily Warren, Diana Ruth Elbourne, Joanna Sturgess, Leonardo Bevilacqua, Jennifer McGowan, Anne Mathiot, Russell M Viner

**Affiliations:** 1 Department of Public Health, Environments and Society, London School of Hygiene and Tropical Medicine, London, UK; 2 Department of Medical Statistics, London School of Hygiene and Tropical Medicine, London, UK; 3 Department of Social Science, UCL Institute of Child Health, London, UK

**Keywords:** adolescent, schools, environment, trial, behaviour

## Abstract

**Background:**

Interventions to modify school environments are effective in promoting young people’s health across outcomes, but mechanisms are poorly understood. We assessed mediation in a trial of the Learning Together intervention, building on the recent publication of results of effectiveness for reducing bullying and benefits across secondary outcomes and generally good implementation fidelity.

**Methods:**

Within a cluster-randomised trial involving 40 English schools, we examined student-reported and staff-reported school climate and student-reported involvement with delinquent peers at 24-month and 36-month follow-up, assessing the reliability of measures and whether these mediated health outcomes at a final follow-up.

**Results:**

Response rates and reliability were good for student-reported but not staff-reported measures. The intervention increased student-reported but not staff-reported-positive school climate but, like effects on student health outcomes, these manifested only at a final follow-up. The intervention reduced student-reported contact with delinquent peers at an interim follow-up. Student-reported potential mediators measured at the interim follow-up were associated with most health outcomes at the final follow-up. Adjustment for student-reported school climate and contact with delinquent peers at the interim follow-up did not reduce the associations between trial arm and our health outcomes.

**Conclusion:**

Despite being constrained by imperfect measures and by the late manifestation of impacts on student-reported school climate undermining ability to assess mediation, our study for the first time provides tentative evidence that mediation of intervention effects via improved climate and disengagement from delinquent peers is plausible. Our study provides the first evidence from a trial that whole-school interventions may work by modifying school environments and student relationships.

**Trial registration number:**

ISRCTN10751359.

## Introduction

There is increasing interest in interventions aiming to promote young people’s health by making overall school environments more health-promoting.^1^ A Cochrane review of ‘health promoting schools’ interventions (with environment, community and curriculum components) reported various benefits, including reducing bullying victimisation, smoking and body mass index, and increasing physical activity.^2^ Another review focused on interventions with environmental and not curriculum components also reported multiple health benefits.^3^

But how do such interventions work? A systematic review of theories of how school environments influence health^4^ concluded that the theory of human functioning and school organisation is the most comprehensive theory of change for such interventions.^5^ This postulates that for young people to choose healthier over riskier behaviours, they must possess the autonomy and ability to reason and form relationships, to make informed, healthy decisions. These capacities are facilitated by student engagement with school: good relationships with teachers; commitment to learning; and sense of belonging and participation in the school community. A refinement of the theory suggests that students lacking such school commitments may engage with delinquent peers and risky behaviours as alternative markers of belonging and identity.^6^ The theory also suggests that schools can increase student commitment by modifying school organisation: distributing authority between staff; promoting good staff–student relationships; integrating academic education and broader student development; and ensuring school culture reflects that of the local community.

While there is some evidence in support of this theory from observational studies of school-level determinants of student health,^7^ such mechanisms remain largely unexamined in intervention studies. The only study that has examined whether the health effects of whole-school interventions are mediated by student commitments to school was the Gatehouse Project. This involved a randomised controlled trial (RCT) of a whole-school intervention delivered in Australian secondary schools. Despite reporting effects on various measures of adolescent health-related risk behaviours, the study found no evidence of effects on student’ attachment to school, suggesting that attachment was not a mediator of health effects or that the measure failed to assess attachment.^8^

**Figure 1:**
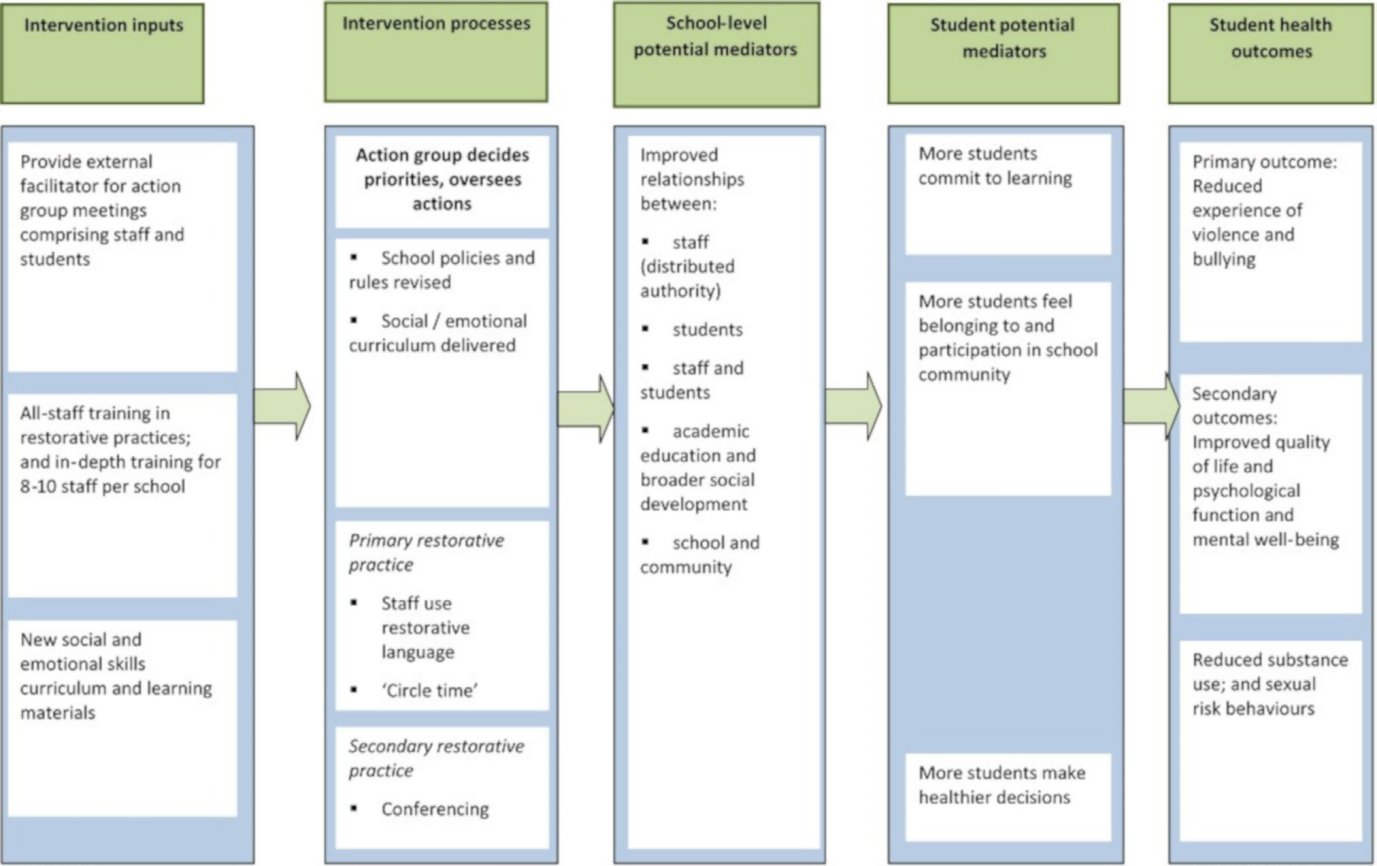
Logic model

We explore these questions in relation to our own recent RCT of the Learning Together intervention.^9^ This whole-school intervention aimed to support schools to implement the restorative practice, staff/student action groups, and a student social and emotional skills curriculum to reduce bullying and aggression, and promote student health across various secondary outcomes. The restorative practice aims to improve relationships to prevent and/or resolve conflicts between students or between staff and students.^10^ It aims to prevent incidents via methods such as ‘circle-time’ (bringing staff and students together to build relationships) and/or resolve incidents via methods such as ‘conferencing’ (bringing together conflicting parties to find ways to avoid further harms). Action groups are school meetings involving diverse students, and senior and junior staff. They coordinate whole-school intervention delivery and review school rules and policies to ensure that these support whole-school change. They aim to build better relationships between the staff and students sitting on the group and signal to the wider student body that the school cares about and intends to act on the views of staff and students to build a supportive school climate.^3^ Social and emotional education aims to ensure that schools teach not only academic knowledge but also attend to students’ broader social development.^11^ Informed by the theory of human functioning and school organisation,^5^ these intervention components were theorised to work synergistically within Learning Together to: distribute decision-making authority across the school; strengthen relationships between and among staff and students; and integrate students’ academic learning and broader development. The intervention did not aim to improve relationships between schools and their local communities, although this might occur as a by-product. These impacts were theorised to transform the whole-school climate and improve staff–student relationships, student commitment to learning and sense of belonging and participation in the school community, thereby reducing student engagement with delinquent peers and risk behaviours, and promoting student health (figure 1).

An RCT of the intervention reported a range of benefits including reducing bullying victimisation, use of tobacco, alcohol and drugs, and contact with the police, and promoting mental well-being, quality of life and psychological functioning. The intervention was found to be implemented with good fidelity although this was much lower for the curriculum component.^12^ This paper aims to examine potential mediators of such effects. We first assess the reliability of our potential mediators. These include existing student-reported measures of views of school climate and engagement with delinquent peers. We also examine a new measure of staff-reported school organisational climate to explore whether intervention effects on the school organisational environment might explain student health outcomes. Our second aim is to assess whether these measures might be mediators of intervention effects on our primary and secondary health outcomes.

## Methods

Here we provide a summary of methods for the trial. For full details, including sample size calculation, see published protocol and trial report.^9 12^ We undertook a two-arm parallel repeat cross-sectional cluster RCT of the Learning Together intervention in 40 secondary schools eligible to take part as state schools in south-eastern England with government inspections rated as requires improvement or above, recruited by the trial team via emails. Our eligible study population consisted of all students: at baseline at the end of year 7 in 2014 (11–12 years); at interim 24-month follow-up; and final 36-month follow-up in 2017. Student data were collected using paper questionnaires in classrooms under examination conditions by trained fieldworkers blind to allocation. Using computer-generated random numbers, schools were then allocated 1:1 to intervention or control stratified by schools: single sex versus mixed sex; school-level student free-school-meal eligibility (0%–23%;>23%) indicating poverty; and General Certificate of Secondary Education results accounting for student baseline attainment (above/below the median score for England of 1000).

All staff in intervention schools received training to implement restorative practices. Around 5–10 staff per school received in-depth training to deliver restorative conferences. All schools were provided with a manual to guide the action group comprising at least six staff and six students, led by a member of the school’s senior leadership team. Groups were supported by external facilitators in the first 2 years of intervention but in the third year, the group was facilitated by school staff only. Action groups aimed to revise rules and policies so that these supported deliveries of restorative practice and coordinate implementation. Schools were provided with lesson plans and slides to guide the delivery of a social and emotional skills curriculum targeting students in years 8–10 who received 5–10 hours of teaching per year. Schools randomised to the control group continued with the normal practice.

Primary outcomes were bullying victimisation measured by the Gatehouse Bullying Scale^8^ and perpetration of aggression measured by the Edinburgh Study of Youth Transitions and Crime Scale^13^ at 36 months. Secondary outcome included use of tobacco, alcohol and drugs, mental well-being, psychological functioning and quality of life and contacts with the police and National Health Service (NHS), as described in our protocol and the main trial paper.^9 12^ The focus of the present paper is on three potential mediator measures, informed by the intervention logic model (figure 1).

The first focused on student reports of school climate, assessed using an established measure, the Beyond Blue School Climate Questionnaire (BBSCQ) scale, which includes 28 student-reported items arranged in four subscales covering: staff–student relationships, student sense of belonging in the school community; student commitment to learning; and student participation at school (online supplementary table S1).^14^ Students were asked to rate their level of agreement with items and their responses scored between 1 (complete disagreement) and 4 (complete agreement). Scores were then averaged within the subscales to obtain the subscale scores, and across all items to obtain the overall BBSCQ score. This measure aligns closely with the key theoretical constructs from the theory of human functioning and school organisation concerning staff–student relationships, student commitment to learning, sense of belonging and participation.^5^

10.1136/jech-2018-211443.supp1 Supplementary data

The second potential mediator focused on student reports of engagement with delinquent peers, assessed by the Young People’s Development Programme single-item measure asking students whether their friends who are the same age as them have been told off, stopped or picked up by the police in the last 12 months.^15^ Data for these two measures were collected via baseline, interim and final follow-up student surveys.

The third potential mediator focused on staff reports of their perception of school organisational climate, using a new scale (online supplementary table S1), which was assessed for the reliability at baseline and amended to include the most reliable items.^16^ This was a 26-item scale with subscales measuring: whether the authority is distributed among staff; staff–student relationships; integration of students’ academic education and broader development; and school–community relationships. Staff were asked to rate their level of agreement with items, with responses scored between 1 (strongly agree) and 4 (strongly disagree). Items were recoded so that a higher score indicated what, from the perspective of our theory of change, would be regarded as a less-healthy school organisational climate. Factor scores were derived within the subscales to obtain the subscale scores and across the subscale scores to obtain the overall score. Data for this measure were collected via structured telephone interviews from staff in intervention and control schools. ‘Baseline’ data were collected in September–November 2014 from one member of each school’s senior leadership team and two other members of staff identified by this individual. Interim follow-up data were then collected in September–November 2017 from one member of each school’s senior leadership team or the staff member leading the action group.

The trial was approved by the UCL (ref 5248/001) and IoE (ref. FCL 566) Research Ethics Committees. Written, informed consent was obtained at school level (head-teacher) for random allocation and intervention, and at the individual-participant level for data collection. Information sheets and consent forms for surveys were identical in intervention and control schools. Parents of students were informed about the study and could withdraw their children from research activities. The trial was prospectively registered as ISRCTN10751359 with the ISRCTN Registry on 30 January 2014.^9^

For potential mediators, completion was assessed in terms of the proportion of items completed by participants and the proportion of participants who completed at least half of the items in a subscale or scale. We then assessed interitem scale reliability using Cronbach’s alpha. We used this rather than ordinal alphas for ordinal scales for simplicity and because Cronbach’s alpha provides suitably conservative estimates for such measures.^17^

We then used a causal steps approach to assess whether our potential mediators might have mediated intervention effects on our primary and secondary outcomes.^18^ Four criteria needed to be met. The first, that the intervention was associated with the outcomes, was examined in our main trial analysis where, as previously stated, we observed a range of statistically significant positive effects. The second criterion required an association between the intervention and the potential mediators. We therefore assessed associations between trial arm and: our student-reported potential mediators measured at interim and final follow-ups; and our staff-reported potential mediator measured at the interim follow-up. These analyses, like those reported on our primary and secondary outcomes, used the intention-to-treat principle that is including all schools and participants in their groups as allocated. Each measure was analysed using a separate mixed model with the outcomes from each time-point treated as a repeated measure outcome. Fixed effects of the arm, follow-up time and the interaction between arm and time were specified, and the estimated baseline measures were constrained to be identical in the two arms of the trial, equivalent to adjusting for baselines. Random effects for schools and participants were specified to allow for correlations within schools and repeated measures within participants. Explicit consideration of potential confounders and how they are controlled for is an important part of statistical mediation analysis. We, therefore, report unadjusted analyses as well as analyses adjusted for baseline measures of the outcomes, sex, ethnicity, socioeconomic status (measured using the Family Affluence Scale)^19^ as well as for the school-level stratifying factors (single-sex versus mixed-sex school; school level deprivation; academic attainment strata). For continuous outcomes, we report unadjusted and adjusted mean differences with 95% CIs and adjusted effect sizes (standardised mean difference). For binary and ordinal outcomes, we report unadjusted and adjusted ORs.

The third criterion required that the potential mediator was associated with the outcomes. We, therefore, examined associations between our potential mediators measured at the interim follow-up, and our primary and secondary outcomes measured at the final follow-up so that we were assured of the temporality of the associations. Adjustment for potential confounders was as per previous analyses. The final criterion required that adjusting for potential mediators reduced the association between the intervention and the outcome measures. Therefore, we assessed the effect of adjusting for our potential mediators measured at the interim follow-up on the associations between trial arm and our primary and secondary outcomes measured at the final follow-up in the fully adjusted analysis. As differences between students who completed both baseline and follow-up surveys and those who only completed baseline were observed, we conducted a sensitivity analysis using multiple imputation by chained equations to impute missing data for participants with incomplete outcome data. All analyses were completed by staff blind to allocation.

## Results

For trial participation and follow-up rates, participant characteristics and overall outcomes by allocation, see the main trial report.^12^ Student data were available from all schools. Student-reported measures of potential mediators had good response rates and reliability with over 85% of all participants completing all items and with multi-item scales and subscales having Cronbach’s alphas of >90% for the overall scale and 70% for the subscales at the baseline and interim follow-up (table 1). Staff data were available from 40 schools at the baseline and 31 schools at the interim follow-up. The staff-reported potential mediator had slightly lower item-response rates, but still >70%. Cronbach’s alpha for the overall staff-reported measure was >80% at the baseline and over 60% at the interim follow-up, with Cronbach’s alphas for the subscales being somewhat lower. There were small differences between students completing all surveys and those completing only baselines, with attrition higher among those from smaller schools or more deprived neighbourhoods, of white British ethnicity, with non-working parents or not living with two biological parents (table 2).

**Table 1:** Mediator measures response and reliability

Measure	Response rates				Internal consistency— Cronbach’s alpha (standardised)	
	Baseline		Interim follow-up		Baseline	Interim
	Completed all items (n)	Completed half or more of items (n)	Completed all items (n)	Completed half or more of items (n)		
**Student view on school climate**						
Overall	5733 85.99%	6635 99.52%	5549 88.22%	6265 99.60%	0.9137	0.9170
Student sense of belonging subscale	6293 94.39%	6613 99.19%	5965 94.83%	6240 99.21%	0.7952	0.8225
Student commitment to academic values subscale	6519 97.78%	6581 98.71%	6190 98.41%	6231 99.06%	0.7394	0.7732
Student perception of supportive teacher relationships subscale	6221 93.31%	6631 99.46%	5935 94.36%	6247 99.32%	0.8804	0.8938
Student perception of participative school environment subscale	6396 95.94%	6600 99.00%	6071 96.52%	6231 99.06%	0.8005	0.8313
**Student report of friends’ contact with police in the last year**	6494 97.41%	NA	6167 98.04%	NA	NA	NA
Staff view on school organisational climate						
Overall	93 77.50%	99 82.50%	31 77.50%	31 77.50%	0.8357	0.6266
Authority distributed among staff subscale	98 81.67%	99 82.50%	31 77.50%	31 77.50%	0.6340	0.5952
Staff relationships with students subscale	98 81.67%	99 82.50%	31 77.50%	31 77.50%	0.7349	0.7633
Integration of students’ academic education and broader social development subscale	98 81.67%	99 82.50%	31 77.50%	31 77.50%	0.6984	0.4839
School–community relationships subscale	96 80.00%	99 82.50%	31 77.50%	31 77.50%	0.7363	0.7536

**Table 2:** Differences in characteristics between students completing all surveys and those completing baseline only

Covariate	Category	Total	Baseline data only
		N (%) n=3337	N (%) n=1040
School size	Small	1574 (47.17)	542 (52.12)
	Large	1763 (52.83)	498 (47.88)
School neighbourhood deprivation	Low score	1696 (47.83)	525 (50.48)
	High score	1741 (52.17)	515 (49.52)
Free school meal eligibility	Low score	1663 (49.84)	527 (50.67)
	High score	1674 (50.16)	513 (49.33)
Student gender	Female	1644 (50.15)	515 (50.54)
	Male	1634 (49.85)	504 (49.46)
Student ethnicity	White British	1383 (41.46)	446 (43.34)
	Other	1921 (58.14)	583 (56.66)
Family structure	Two parents	2388 (71.97)	647 (62.75)
	Other	930 (28.03)	384 (37.25)
Parental working	Not in work	287 (10.57)	115 (14.32)
	In work	2429 (89.43)	688 (85.68)
Family affluence	Low affluence	1194 (36.81)	371 (36.88)
	High affluence	2050 (63.19)	365 (63.12)

The intervention had statistically significant effects on potential mediators, being associated with increased student-reported-positive school climate as well as its constituent subscales at the final but not the interim follow-up. The intervention was also significantly associated with a decrease in student-reported measure of involvement with delinquent peers at the interim follow-up but one slightly short of statistical significance at the final follow-up (table 3). The intervention had no statistically significant effects on the overall staff-reported measure of school organisational climate or its constituent subscales measured at the interim follow-up.

**Table 3:** Intervention effects on mediators at the interim and final follow-ups

Measure	Interim follow-up						Final follow-up					
	Arm		Unadjusted effect		Adjusted effect		Arm		Unadjusted effect		Adjusted effect	
	Control Mean (SE)	Intervention Mean (SE)	Difference (95% CI)	P value	Difference (95% CI)	P value	Control Mean (SE)	Intervention Mean (SE)	Difference (95% CI)	P value	Difference (95% CI)	P value
**Student view of school climate**												
Overall	2.92 (0.03)	2.90 (0.03)	−0.00 (−0.02 to 0.02)	0.915	−0.00 (−0.02 to 0.02)	0.993	2.82 (0.03)	2.85 (0.03)	0.05 (0.03 to 0.07)	<0.001	0.05 (0.03 to 0.08)	<0.001
Student perception of supportive teacher relationships subscale	2.76 (0.04)	2.70 (0.04)	−0.03 (−0.06 to 0.00)		−0.02 (−0.05 to 0.01)		2.64 (0.03)	2.66 (0.03)	0.05 (0.02 to 0.08)		0.06 (0.03 to 0.09)	
Student sense of belonging	2.84 (0.03)	2.88 (0.03)	0.04 (0.01 to 0.07)		0.04 (0.01 to 0.07)		2.78 (0.02)	2.84 (0.02)	0.06 (0.03 to 0.09)		0.06 (0.03 to 0.09)	
Student perception of participative school environment subscale	2.96 (0.03)	2.91 (0.03)	−0.02 (−0.05 to 0.01)		−0.03 (−0.06 to 0.01)		2.81 (0.04)	2.82 (0.04)	0.05 (0.02 to 0.08)		0.05 (0.01 to 0.08)	
Student commitment to academic values subscale	3.51 (0.01)	3.53 (0.01)	0.00 (−0.02–0.03)		0.00 (−0.02 to 0.03)		3.42 (0.01)	3.46 (0.01)	0.03 (0.01 to 0.06)		0.03 (0.01 to 0.06)	
**Staff view of school organisational climate**												
Overall^*^	−0.13 (0.14)	−0.05 (0.29)	0.08 (−0.46 to 0.63)	0.766	0.19 (−0.35 to 0.73)	0.499						
Authority distributed among staff subscale	2.04 (0.11)	2.49 (0.20)	0.45 (0.05 to 0.85)		0.39 (−0.10 to 0.88)							
Staff relationships with students subscale	3.04 (0.14)	2.94 (0.23)	−0.10 (−0.58 to 0.38)		−0.22 (−0.80 to 0.35)							
Integration of students’ academic education and broader social development subscale	2.07 (0.10)	1.65 (0.09)	−0.43 (−0.70 to −0.15)		−0.27 (−0.55 to 0.01)							
School–community relationships subscale	1.98 (0.13)	2.07 (0.20)	0.08 (−0.35 to 0.52)		−0.10 (−0.59 to 0.40)							

Measure	Interim follow-up						Final follow-up					
	Arm		Unadjusted effect		Adjusted effect		Arm		Unadjusted effect		Adjusted effect	
	Control N (%)	Intervention N (%)	OR (95% CI)	P value	OR (95% CI)	P value	Control N (%)	Intervention N (%)	OR (95% CI)	P value	OR (95% CI)	P value
**Student report of friends’ contact with police in the last year**												
No	2203 (70.05)	2271 (75.15)	0.80 (0.66 to 0.97)	0.024	0.80 (0.66 to 0.96)	0.020	2073 (68.64)	2044 (73.21)	0.84 (0.69 to 1.02)	0.074	0.83 (0.69 to 1.01)	0.066
Yes	942 (29.95)	751 (24.85)					947 (31.36)	748 (26.79)				

*The  overall score is a weighted sum of subscales; its range is therefore expected to differ from those of the subscales  on  which it is based.

Student reports of school climate and of friends having contact with police measured at the interim follow-up were associated with primary health outcome measures as well as with secondary health outcome measures (other than age of sexual debut and use of contraception at last sexual intercourse) at the final follow-up in unadjusted and adjusted analyses (table 4). Staff reports of school organisational climate measured at the interim follow-up were not associated with any primary or secondary outcomes at the final follow-up in unadjusted or adjusted analyses, other than for one unadjusted association between school organisational climate and students’ use of NHS in the past 12 months, which disappeared on adjustment.

**Table 4:** Effects of potential mediators at interim follow-up on outcomes at the final follow-up

**Continuous student outcomes**	**Student view of school climate**				**Friends’ contact with police in last year**				**Staff view of school organisational climate**			
	**Unadjusted effect**		**Adjusted effect**		**Unadjusted effect**		**Adjusted effect**		**Unadjusted effect**		**Adjusted effect**	
	**Difference (95% CI)**	**P value**	**Difference (95% CI)**	**P value**	**Difference** **(95% CI)**	**P value**	**Difference** **(95% CI)**	**P value**	**Difference** **(95% CI)**	**P value**	**Difference** **(95% CI)**	**P value**
Bullying victimisation (GBS overall score)	−0.20 (−0.24 to −0.17)	<0.001	−0.22 (−0.25 to −0.18)	<0.001	0.12 (0.08 to 0.15)	<0.001	0.12 (0.09 to 0.16)	<0.001	−0.01 (−0.04 to 0.04)	0.617	0.00 (−0.03 to 0.04)	0.841
Aggression perpetration (ESYTC overall score)	−2.49 (−2.87 to −2.11)	<0.001	−2.57 (−2.96 to −2.18)	<0.001	2.73 (2.34 to 3.11)	<0.001	2.72 (2.32 to 3.11)	<0.001	−0.20 (−0.59 to 0.19)	0.317	0.07 (−0.36 to 0.50)	0.742
Quality of life (PedsQL overall score)	5.81 (4.84 to 6.77)	<0.001	5.84 (4.87 to 6.81)	<0.001	−2.03 (−3.00 to −1.07)	<0.001	−2.40 (−3.36 to −1.44)	<0.001	−0.50 (−1.58 to 0.59)	0.372	0.29 (−0.70 to 1.27)	0.568
Psychological functioning (SDQ total difficulties score	−3.17 (−3.54 to −2.79	<0.001	−3.21 (−3.59 to −2.83)	<0.001	1.17 (0.80 to 1.55)	<0.001	1.31 (0.93 to 1.69)	<0.001	0.01 (−0.39 to 0.41)	0.967	0.06 (−0.33 to 0.45)	0.773
SWEMWBS total well-being index)	3.83 (3.44 to 4.22)	<0.001	3.91 (3.51 to 4.31)	<0.001	−0.71 (−1.12 to −0.31)	0.001	−0.81 (−1.22 to −0.40)	<0.001	0.12 (−0.28 to 0.53)	0.555	0.23 (−0.19 to 0.64)	0.284
Health utility (CHU9D overall score)	0.06 (0.05 to 0.06)	<0.001	0.06 (0.05 to 0.07)	<0.001	−0.02 (−0.03 to −0.01)	<0.001	−0.02 (−0.03 to −0.01)	<0.001	−0.00 (−0.01 to 0.00)	0.387	0.00 (−0.01 to 0.01)	0.583
Age of sexual debut	−0.16 (−1.04 to 0.72)	0.724	0.09 (−0.81 to 1.00)	0.837	0.61 (−0.22 to −1.43)	0.149	0.46 (−0.37 to 1.29)	0.281	−0.46 (−1.41 to 0.50)	0.348	−0.95 (−2.02 to 0.12)	0.082
Bullying perpetration (MAS bullying subscale score)	−1.54 (−1.75 – −1.33)	<0.001	−1.53 (−1.74 to −1.33)	<0.001	1.55 (1.34 to 1.77)	<0.001	1.46 (1.25 to 1.67)	<0.001	−0.13 (−0.58 to 0.31)	0.559	0.10 (−0.20 to 0.39)	0.517

**Categorical student outcomes**	**OR** **(95% CI)**	**P value**	**OR** **(95% CI)**	**P value**	**OR** **(95% CI)**	**P value**	**OR** **(95% CI)**	**P value**	**OR** **(95% CI)**	**P value**	**OR** **(95% CI)**	**P value**
Ever smoked	0.42 (0.35 to 0.51)	<0.001	0.40 (0.33 to 0.49)	<0.001	3.89 (3.24 to 4.66)	<0.001	4.12 (3.41 to 4.97)	<0.001	0.92 (0.67 to 1.26)	0.608	0.96 (0.71 to 1.30)	0.801
Ever drunk alcohol	0.46 (0.39 to 0.55)	<0.001	0.44 (0.37 to 0.53)	<0.001	2.63 (2.22 to 3.11)	<0.001	2.69 (2.25 to 3.22)	<0.001	0.94 (0.60 to 1.48)	0.803	1.08 (0.82 to 1.41)	0.587
Ever been really drunk	0.62 (0.50 to 0.76)	<0.001	0.58 (0.47 to 0.71)	<0.001	1.86 (1.54 to 2.24)	<0.001	2.03 (1.67 to 2.47)	<0.001	0.91 (0.70 to 1.18)	0.468	0.95 (0.73 to 1.25)	0.722
Ever been offered illicit drugs	0.36 (0.31 to 0.42)	<0.001	0.33 (0.29 to 0.39)	<0.001	3.87 (3.37 to 4.44)	<0.001	3.91 (3.39 to 4.51)	<0.001	1.05 (0.81 to 1.35)	0.715	1.08 (0.85 to 1.38)	0.532
Used contraception at last sexual intercourse	1.69 (1.00 to 2.86)	0.049	2.22 (1.21 to 4.07)	0.010	1.38 (0.83 to 2.31)	0.215	1.25 (0.72 to 2.18)	0.435	0.70 (0.48 to 1.02)	0.064	0.76 (0.44 to 1.29)	0.311
Use of NHS in past 12 months	0.80 (0.70 to 0.92)	0.002	0.79 (0.68 to 0.92)	0.002	1.45 (1.26 to 1.68)	<0.001	1.42 (1.22 to 1.64)	<0.001	0.87 (0.78 to 0.97)	0.014	0.89 (0.79 to 0.99)	0.037
Contact with police	0.39 (0.32 to 0.49)	<0.001	0.37 (0.29 to 0.46)	<0.001	5.08 (4.15 to 6.22)	<0.001	5.09 (4.13 to 6.28)	<0.001	0.80 (0.64 to 0.99)	0.037	0.86 (0.72 to 1.03)	0.111

CHU9D, Chuld Health Utility 9D;  ESYTC, Edinburgh Study of Youth Transitions and Crime; GBS, Gatehouse Bullying Scale; MAS, Modified Aggression Scale;  NHS,  National Health Service; PedsQL, Pediatric Quality of Life Inventory; SDQ, Strengths and Difficulties Questionnaire; SWEMWBS, Short Warwick Edinburgh Mental Wellbeing Scale;.

Our analysis of the effect of additionally adjusting for potential mediators measured at the interim follow-up on the associations previously found between the intervention and primary and secondary health outcomes measured at the final follow-up found no evidence that this adjustment made any difference except marginally in the case of the intervention effect on well-being, where adjustment for both student-reported school climate and friends’ contact with the police removed the previously statistically marginal intervention effect at the final follow-up (table 5).

**Table 5:** Intervention effects on student primary and secondary outcomes at the final follow-up before and after adjusting for potential mediators at the interim follow-up

**Continuous student outcomes**	**Arm**		**Unadjusted effect**		**Adjusted effect** **(baseline covariates)**		**Adjusted effect** **(baseline covariates plus student view of school climate)**		**Adjusted effect** **(baseline covariates plus friends’ contact with police in last year)**		**Adjusted effect** **(baseline covariates plus staff view of school organisational climate)**	
	**Control** **Mean (SE)**	**Intervention** **Mean (SE)**	**Difference (95% CI)**	**P value**	**Difference (95% CI)**	**P value**	**Difference** **(95% CI)**	**P value**	**Difference** **(95% CI)**	**P value**	**Difference** **(95% CI)**	**P value**
Bullying victimisation (GBS overall score)	0.34 (0.02)	0.29 (0.02)	−0.03 (−0.06 to −0.00)	0.039	−0.03 (−0.06 to −0.00)	0.044	−0.04 (−0.08 to −0.01)	0.007	−0.04 (−0.07 to −0.01)	0.016	−0.02 (−0.07 to 0.03)	0.380
Aggression perpetration (ESYTC overall score)	4.33 (0.20)	4.04 (0.21)	−0.07 (−0.38 to 0.25)	0.684	−0.13 (−0.43 to 0.18)	0.421	−0.20 (−0.52 to 0.13)	0.229	−0.12 (−0.44 to 0.20)	0.469	0.03 (−0.58 to 0.64)	0.917
Quality of life (PedsQL overall score)	78.82 (0.54)	80.65 (0.55)	1.16 (0.41 to 1.90)	0.002	1.44 (0.70 to 2.17)	<0.001	1.42 (0.64 to 2.21)	<0.001	1.26 (0.49 to 2.03)	0.001	1.43 (0.09 to 2.77)	0.036
Psychological functioning (SDQ total difficulties score	12.20 (0.18)	11.51 (0.19)	−0.51 (−0.80 to −0.22)	<0.001	−0.54 (−0.83 to −0.25)	<0.001	−0.62 (−0.92 to −0.31)	<0.001	−0.54 (−0.85 to −0.24)	<0.001	−0.38 (−0.92 to 0.17)	0.170
SWEMWBS total well-being index)	22.88 (0.19)	23.32 (0.19)	0.27 (−0.06 to 0.60)	0.115	0.33 (0.00 to 0.66)	0.048	0.27 (−0.08 to 0.62)	0.141	0.23 (−0.13 to 0.58)	0.210	0.21 (−0.36 to 0.80)	0.463
Health utility (CHU9D overall score)	0.85 (0.00)	0.86 (0.01)	0.01 (−0.00 to 0.01)	0.244	0.01 (−0.00 to 0.01)	0.080	0.00 (−0.00 to 0.01)	0.153	0.00 (−0.00 to 0.01)	0.217	0.00 (−0.01 to 0.01)	0.570
Age of sexual debut	13.11 (0.43)	12.54 (0.49)	−0.58 (−1.97 to 0.81)	0.416	−0.35 (−1.48 to 0.78)	0.541	−0.24 (−1.48 to 1.00)	0.703	−0.40 (−1.65 to 0.84)	0.525	−0.51 (−2.03 to 1.01)	0.513
Bullying perpetration (MAS bullying subscale score)	2.75 (0.21)	2.33 (0.21)	−0.28 (−0.84 to 0.29)	0.334	−0.26 (−0.57 to 0.05)	0.097	−0.29 (−0.57 to −0.00)	0.047	−0.16 (−0.45 to 0.13)	0.279	−0.27 (−0.69 to 0.14)	0.193

**Categorical student outcomes**	**Control** **n (%)**	**Intervention** **n (%)**	**OR** **(95% CI)**	**P value**	**OR** **(95% CI)**	**P value**	**OR** **(95% CI)**	**P value**	**OR** **(95% CI)**	**P value**	**OR** **(95% CI)**	**P value**
Ever smoked												
No	2293 (77.70)	2318 (84.17)	0.59 (0.43 to 0.81)	0.001	0.58 (0.43 to 0.80)	0.001	0.55 (0.39 to 0.78)	0.001	0.57 (0.40 to 0.81)	0.002	0.65 (0.44 to 0.97)	0.037
Yes	658 (22.30)	436 (15.83)										
Ever drunk alcohol												
No	1677 (56.43)	1735 (62.43)	0.75 (0.58 to 0.79)	0.029	0.72 (0.56 to 0.92)	0.009	0.72 (0.55 to 0.95)	0.019	0.74 (0.56 to 0.97)	0.028	0.76 (0.53 to 1.09)	0.134
Yes	1295 (43.57)	1044 (37.57)										
Ever been really drunk												
No	788 (53.14)	721 (61.21)	0.50 (0.29 to 0.87)	0.014	0.47 (0.31 to 0.71)	<0.001	0.42 (0.27 to 0.65)	<0.001	0.47 (0.31 to 0.71)	<0.001	0.66 (0.47 to 0.93)	0.019
Yes	695 (46.86)	457 (38.79)										
Ever been offered illicit drugs												
No	1913 (64.41)	1997 (72.54)	0.52 (0.34 to 0.79)	0.002	0.51 (0.36 to 0.73)	<0.001	0.44 (0.30 to 0.64)	<0.001	0.51 (0.36 to 0.73)	<0.001	0.70 (0.50 to 0.96)	0.028
Yes, but did not try	744 (25.05)	567 (20.60)										
Yes, and tried them	313 (10.54)	189 (6.87)										
Used contraception at last sexual intercourse												
No	64 (23.10)	36 (21.95)	1.18 (0.56 to 2.48)	0.658	1.08 (0.50 to 2.35)	0.841	1.60 (0.65 to 3.91)	0.305	1.34 (0.56 to 3.22)	0.511	1.40 (0.64 to 3.06)	0.405
Yes	213 (76.90)	128 (78.05)										
Use of NHS in past 12 months												
No	1605 (53.22)	1472 (52.59)	0.96 (0.83 to 1.12)	0.639	0.96 (0.82 to 1.11)	0.565	0.96 (0.81 to 1.13)	0.600	0.99 (0.84 to 1.17)	0.916	0.96 (0.82 to 1.13)	0.613
Yes	1411 (46.78)	1327 (47.41)										
Contact with police												
No	2626 (86.52)	2485 (88.43)	0.75 (0.57 to 0.99)	0.041	0.74 (0.56 to 0.97)	0.027	0.66 (0.49 to 0.88)	0.004	0.72 (0.54 to 0.95)	0.022	0.94 (0.73 to 1.21)	0.641
Yes	409 (13.48)	325 (11.57)										

CHU9D, Chuld Health Utility 9D NHS National Health Service; ESYTC, Edinburgh Study of Youth Transitions and Crime; GBS, Gatehouse Bullying Scale;  PedsQL, Pediatric Quality of Life Inventory; SDQ, Strengths and Difficulties Questionnaire; SWEMWBS, Short Warwick Edinburgh Mental Wellbeing Scale; MAS, Modified Aggression Scale;

The multiple imputation analysis produced results, available on request, which did not differ from the main analysis in the pattern, size or statistical significance of the associations found.

## Conclusion

### Summary of key findings

The student-reported measures of potential mediators had good response rates and reliability. The staff-reported measure had somewhat lower response rates and interitem reliability. The intervention appeared to impact on: student perceptions of school climate but these (like intervention effects on primary and secondary student health outcomes) did not manifest until the final follow-up; and student contact with delinquent peers at the interim follow-up. The student-reported potential mediators measured at the interim follow-up were associated with most student health outcomes. Adjustment for student-reported school climate and contact with delinquent peers at the interim follow-up did not affect associations between intervention and health outcomes.

### Study limitations

The study used a well-established, multi-item measure of student views of school climate drawing on date from all students completing surveys. However, the study used a weaker, single-item measure of student involvement with delinquent peers and used a new and less reliable staff-reported measure of school organisational climate which drew on very small samples of staff (three individuals at baseline and one individual at the interim follow-up). Therefore, our ability to assess whether student contact with delinquent peers and staff-reported organisational climate acted as potential mediators of intervention effects on student health may have been limited. The RCT included the interim follow-up at 24 months and the final follow-up at 36 months. Intervention effects on primary and secondary health outcomes as well as effects on student views of school climate manifested only at the latter time-point. This meant that our ability to determine definitely whether intervention effects on school climate might mediate effects on student health outcomes was limited. The number of statistical tests might have introduced some false positive results, but we hope that being focused on prior hypotheses limited bias.

### Implications for research and policy

Our study provides the first evidence from an RCT that well-implemented whole-school interventions may be effective both in promoting student health and in improving student relationships with teachers, and sense of commitment, belonging and participation at school. Despite being constrained in its ability to assess mediation by the late manifestation of impacts on student views of school, our study suggests that mediation of intervention effects via these factors is at least plausible, in that the intervention had effects on student-reported potential mediators, which were associated with student health outcomes. The lack of evidence for mediation for the staff-reported measure of school organisational climate may have reflected the poor reliability of this measure. The fact that implementation fidelity was stronger for restorative practice and action groups rather than the curriculum further suggests that any impacts on health outcomes were likely to be achieved through changes to school environment rather than through changes to individual student health-related knowledge and skills. Further research is therefore required to investigate these matters with a better measure of school organisation and longer time scales between measurements. Nonetheless, our study offers tentative evidence that whole-school interventions might work by modifying the school climate as predicted by the theory of human functioning and school organisation.^5^ Regarding generalisability, our trial was carried out in a representative sample of schools in and around London. Our process evaluation identified no factors that might suggest that implementation or effects would be different in other English schools.

**What is already known on this subject**

- Young people’s health can be improved by ‘whole-school’ interventions that aim to render schools more engaging, participative and inclusive.
- But previous evaluations have not examined mediators to assess how such interventions might work.

**What this study adds**

- We found that as well as improving a wide range of health outcomes, our intervention also improved student reports of an engaging, participative and inclusive school climate, and reduced reports of students having delinquent friends. These factors were themselves associated with health outcomes.
- This suggests whole-school interventions might work by engaging young people with school and reducing engagement with pro-risk peers.
