# Supplementary material for: Examining intervention mechanisms of action using mediation analysis within a randomised trial of a whole-school health intervention
Source: J Epidemiol Community Health. 2019 Feb 5;73(5):455–64. doi: 10.1136/jech-2018-211443 (PMC6581112; doi:10.1136/jech-2018-211443)
Supplement: Supplementary data [file jech-2018-211443supp001.pdf]

Table S1: Measures of school climate:

**Scale - subscale**/items	**Source**
**Beyond Blue School Climate Questionnaire**	
**Teacher-student relationships**	
My teachers are fair in dealing with students	Manitoba School Improvement Survey[1]
There's at least one teacher or other adult in this school I can talk to if I have a problem	Psychological Sense of School Membership[2]
I feel I can go to my teacher with the things that are on my mind	Quality of School Life[3]
In this school, teachers believe all students can learn	Patterns of Adaptive Learning Survey[4]
In this school, students' ideas are listened to and valued	
In this school, teachers and students really trust one another	
In this school, teachers treat students with respect	
This school really cares about students as individuals	
Most of my teachers really listen to what I have to say	Quality of School Life[3]
Thinking of my teachers this term, I really like:	
**Student sense of belonging in school community**	
I feel very different from most other students here	Psychological Sense of School Membership[2]
I can really be myself at this school	
Other students in this school take my opinions seriously	
I am encouraged to express my own views in my class(es)	Beyond Blue Schools Research Initiative project investigators[5]
Most of the students in my class(es) enjoy being together	
Most of the students in my class(es) are kind and helpful	
Most other students accept me as I am	
I feel I belong at this school	Patterns of Adaptive Learning Survey[4]
**Student commitment to learning**	
I try hard in school	Gatehouse Project Adolescent Health Survey[6]
Doing well in school is important to me	
Continuing or completing my education is important to me	
I feel like I am successful in this school	Patterns of Adaptive Learning Survey[4]
**Student active participation at school**	
There are lots of chances for students at my school to get involved in sports, clubs and other activities outside class	Gatehouse Project Adolescent Health Survey[6]
Teachers notice when students are doing a good job and let them know about it	
At my school, students have a lot of chances to help decide and plan things like school activities, events and policies	
Student activities at this school offer something for everyone	Manitoba School Improvement Survey[1]
Students have a say in decisions affecting them at this school	
Students at this school are encouraged to take part in activities, programs and special events	Beyond Blue Schools Research Initiative Project Investigators[5]
**Staff view on school organisation climate: new scale**	
**Authority distributed among staff**	
The head teacher takes most of the decisions with little staff consultation	

Teachers participate on a regular basis in the development of school policies	Avon Longitudinal Study of Parents and Children head teacher questionnaire[7]
The senior leadership team consult with staff when making decisions	New question
Teachers in this school have a sense of collective responsibility for student learning	The Impact of School Leadership on Pupil Outcomes Key Staff Questionnaire – Secondary Schools[8]
Teachers in this school have a sense of collective responsibility for student wellbeing	
Teachers and other staff in the classroom work collaboratively	
**Staff relationships with students**	
In my school students participate in decision making	Adapted from The Impact of School Leadership on Pupil Outcomes Key Staff Questionnaire – Secondary Schools[8]
Teachers in this school always show respect towards students	
Students' views are listened to and taken seriously by staff in this school	Avon Longitudinal Study of Parents and Children head teacher questionnaire[8]
Teaching strategies at this school enable students to build their own knowledge	Adapted from The Impact of School Leadership on Pupil Outcomes Key Staff Questionnaire – Secondary Schools[8]
There are opportunities for students to take responsibilities for their own learning in school	
In this school the senior leadership team makes decisions without consulting students	New question
Teachers at this school are often involved in extracurricular activities	Adapted from Avon Longitudinal Study of Parents and Children head teacher questionnaire[7]
In my school teachers mix with students at break times	New question
In my school teachers mix with students at lunch time	
In my school, teachers avoid intervening in students disputes outside the classroom	
**Integration of students' academic education and broader social development**	
The school has a system for rewarding students who achieve in non-academic areas e.g. sport, arts	Adapted from Avon Longitudinal Study of Parents and Children head teacher questionnaire[7]
Our school provides a broad range of extracurricular activities for students (e.g. plays, athletics, music, dance)	The Impact of School Leadership on Pupil Outcomes Key Staff Questionnaire – Secondary Schools[8]
The school development/improvement plan has targets related to student health and wellbeing	Adapted from School Health Research Network school questionnaire[9]
School INSET/training days often focus on student health	
The school has a comprehensive written policy to address student smoking, drugs or alcohol use	
The school teaches a social and emotional learning curriculum	
**School-community relationships**	
Parents often visit the school	The Impact of School Leadership on Pupil Outcomes Key Staff Questionnaire – Secondary Schools[8]

This school engages parents in school improvement efforts	Adapted from The Impact of School Leadership on Pupil Outcomes Key Staff Questionnaire – Secondary Schools[8]
This school aims to build community support for the school's improvement efforts	
Parents give a lot of support to the work of the school	Avon Longitudinal Study of Parents and Children head teacher questionnaire[7]

[1] Earl L, Torrance N, Sutherland S, et al. Manitoba School Improvement Program Final Evaluation Report. Toronto Ontario Institute for Studies in Education; 2003.
[2] Goodenow C. The psychological sense of school membership among adolescents: scale development and educational correlates. Journal of Early Adolescence 1993;13:21-43.
[3] Epstein JL, McPartland JM. The concept and measurement of the quality of school life American Educational Research Journal 1976;13(1):15-30.
[4] Roeser RW, Midgley C, Urdan TC. Perceptions of the school psychological environment and early adolescents' psychological and behavioral functioning in school: the mediating role of goals and belonging. Journal of Educational Psychology 1996;88(3):408-22.
[5] Sawyer MG, Pfeiffer S, Spence SH, et al. School-based prevention of depression: a randomized controlled study of the Beyond Blue schools research initiative. Journal of Child Psychology and Psychiatry 2010;51(2):199-209.
[6] Bond L, Patton G, Glover S, et al. The Gatehouse Project: can a multilevel school intervention affect emotional wellbeing and health risk behaviours? Journal of Epidemiology and Community Health 2004;58(12):997-1003.
[7] Avon Longitudinal Study of Parents and Children. Questionnaire for the Head teacher http://www.bristol.ac.uk/alspac/researchers/resources-available/data-details/questionnaires/documents/ques-s07-questionnaire-for-the-head-teacher.pdf. Bristol: University of Bristol; 2002.
[8] Day C, Sammons P, Hopkins D, et al. The Impact of School Leadership on Pupil Outcomes Interim Report. London: Department for Education; 2007.
[9] Centre for the Development and Evaluation of Complex Interventions for Public Health Improvement. Schools Health Research Network http://man301110a.decipher.uk.net/en/content/cms/research/research-projects/shrn/. Cardiff: DECIPHer; 2014.
